# Leaf resistance to *Botrytis cinerea* in wild tomato *Solanum habrochaites* depends on inoculum composition

**DOI:** 10.3389/fpls.2023.1156804

**Published:** 2023-08-02

**Authors:** Yaohua You, Iván Astudillo-Estévez, Bert Essenstam, Si Qin, Jan A. L. van Kan

**Affiliations:** ^1^ Laboratory of Phytopathology, Wageningen University, Wageningen, Netherlands; ^2^ Wageningen University & Research, Unifarm, Wageningen, Netherlands

**Keywords:** grey mould, incompatible interaction, resistance, *Solanum*, wild tomato

## Abstract

Tomato (S*olanum lycopersicum*) cv. Moneymaker (MM) is very susceptible to the grey mould *Botrytis cinerea*, while quantitative resistance in the wild species *Solanum habrochaites* (accession LYC4) has been reported. In leaf inoculation assays, an effect of nutrient and spore concentration on disease incidence was observed. Resistance in LYC4 leaves was manifested as a high incidence of tiny black, dispersed spots which did not expand (“incompatible interaction”) and was pronounced when *B. cinerea* was inoculated at high spore density (1000 spores/µL) in medium with 10 mM sucrose and 10 mM phosphate buffer. Under the same condition, a high frequency of expanding lesions was observed on MM leaves (“compatible interaction”). Remarkably, inoculation of LYC4 with a high spore density in medium with higher concentrations of sucrose and/or phosphate as well as lower spore density (30 spores/µL) in medium with low sucrose and phosphate, all resulted in a higher percentage of expanding lesions. The lesion sizes at 3 days post inoculation differed markedly between all these inoculation conditions. This inoculation method provides a convenient tool to study mechanisms that determine the distinction between compatible and incompatible interactions between *B. cinerea* and a host plant.

## 1 Introduction

The grey mould fungus, *Botrytis cinerea* is a necrotrophic pathogenic fungus with a wide host range. It can infect >1400 plant species (Fillinger and Elad, 2016), including many dozens of important food crops and ornamental plants, mainly in temperate climate regions. Among the cash crops affected most by grey mould are rose, gerbera, tomato, grapevine and strawberry. The damage that grey mould inflicts worldwide on producers, traders and consumers exceeds €2 x 10^9^ per year (Dean et al., 2012).

*B. cinerea* inoculation under laboratory conditions, as commonly performed to study its interaction with host plants, requires a carbon source and phosphate in the inoculum to enable infection (van Den Heuvel, 1981). Gamborg’s B5 basal salt mixture (GB5) supplemented with sucrose or glucose and potassium phosphate has been used to establish consistent infection on detached tomato leaves under lab conditions (Benito et al., 1998). When inoculating leaves of tomato cultivar Moneymaker (MM), serving as a susceptible control, 10 mM sucrose and 10 mM potassium phosphate was sufficient to cause synchronized and reproducible occurrence of expanding lesions (Benito et al., 1998). By contrast, the same inoculation conditions on wild tomato, such as *S. lycopersicoides*, most frequently resulted in non-expanding lesions which displayed as small black spots under the inoculation droplet (Guimarães et al., 2004). Besides, conidia applied on the leaves of a tomato abscisic acid (ABA)-deficient mutant with 10 mM glucose also barely developed expanding lesions but rather triggered the formation of black spots that did not expand. However, the frequency of brownish expanding lesions increased either by the addition of potassium phosphate or by providing higher concentrations of glucose in the inoculum (Audenaert et al., 2002). How the addition of sugars or phosphates can facilitate *B. cinerea* infection on tomato leaves is not well understood.

Except for the effect of nutrient supplements, the impact of spore density in the inoculum on the infection outcome has not been experimentally studied in *B. cinerea*. In most published experiments, densities ranging from 100 to 1000 spores per µL were used for *B. cinerea* inoculation. It has been proposed that the initial spore density of *B. cinerea* infection might determine the amplitude of fungal attack on the host and the subsequent plant defense responses (Veloso and van Kan, 2018). The fungal pathogen *Plectosphaerella cucumerina* has been reported to display either hemibiotrophic or necrotrophic behaviour, depending on the initial spore density (Pétriacq et al., 2016). Inoculation with high spore densities induced Jasmonic acid-dependent defense responses indicating the infection is recognized as attack of a necrotrophic pathogen. By contrast, low spore density inoculation induced salicylic acid-mediated defenses which are generally activated by hemibiotrophic pathogens (Pétriacq et al., 2016). Also for *B. cinerea*, it should be considered that the initial spore density in the inoculum might play an important role in infection and affect plant defenses in a complex way.

Production of reactive oxygen species (ROS), including hydrogen peroxide (H_2_O_2_) plays an important role during host-microbe interactions. It is a key regulator in defense responses because it participates in the expression of defense-related genes, cell wall fortification and in the host programmed cell death response. Besides, ROS are toxic to microbes and *B. cinerea* can cope with oxidative stress by secreting enzymes functioning in ROS scavenging including catalases and peroxidases during plant infection (Siegmund and Viefhues, 2015). However, as a necrotrophic pathogen, *B. cinerea* is generally assumed to benefit from a host cell death response to enable lesion growth and therefore, ROS production has been suggested to stimulate *B. cinerea* infection (Elad, 1992; Govrin and Levine, 2000). Suppression of plant-generated ROS during *B. cinerea* infection through infiltration of diphenyleneiodonium (DPI), an inhibitor of NADPH oxidase involved in ROS production in *Arabidopsis* leaves, significantly confined lesion expansion (Govrin and Levine, 2000). On the other hand, enhancement of ROS generation in plant tissues resulting from infiltration of mixtures either containing xanthine oxidase plus xanthine or of glucose oxidase plus glucose, gave rise to larger lesions upon *B. cinerea* inoculation (Govrin and Levine, 2000). However, the view that ROS generation is by definition beneficial for *B. cinerea* was refuted by a study from Asselbergh et al. (2007). Upon inoculation on a *S. lycopersicum* ABA-deficient mutant named “*sitiens”* with high levels of resistance to *B. cinerea*, earlier and stronger ROS production coupled with cell wall fortification and protein cross-linking was observed in the epidermal cells. Moreover, application of ascorbate or DPI impaired the ROS burst and compromised the resistance in *sitiens* (Asselbergh et al., 2007). Based on the available evidence from different infection systems, it seems that a ROS burst can play a dual role. On the one hand, induction of a ROS burst is required for host cell death and thereby promotes lesion expansion especially at later stages, while on the other hand, the timely and abundant production of ROS and the subsequent plant defenses in the early stage can efficiently block the fungal invasion. Above all, these observations indicate that the exact role of the host plant ROS burst in resistance against *B. cinerea* needs to be further studied.

Screening of a collection of *S. lycopersicum* genotypes and wild *Solanum* relatives by ten Have et al. (2007) revealed that *S. habrochaites* accession LYC4 displays partial resistance to *B. cinerea* both in leaf and stem inoculation assays. Analysis of recombinant introgression lines from a cross between *S. habrochaites* LYC4 and *S. lycopersicum* MM identified a total of ten quantitative trait loci (QTLs) for resistance (Finkers et al., 2007a; Finkers et al., 2007b), however many of the QTLs represented large genomic regions. In an effort to unravel the physiological mechanisms that contribute to leaf resistance of *S. habrochaites* LYC4, we performed leaf inoculations in different conditions and followed symptom development. Here, we describe that the composition of the inoculation medium and the fungal spore density all contributed to determining whether the LYC4 leaf tissue was resistant or not.

## 2 Materials and methods

### 2.1 Growing S*olanum lycopersicum* and *Solanum habrochaites*

S*olanum lycopersicum* cv. Moneymaker (MM) and the wild species *Solanum habrochaites* (genotype LYC4; ten Have et al., 2007) were grown in potting soil. Plants were grown in a greenhouse at alternating temperatures (21°C day; 19°C night) and a relative humidity of 60%. Plants were exposed for three hours (h)/day to ultraviolet-B (UV-B) light (Philips TL 40W/01 RS) placed at a height of ~1.5m above the table to prevent intumescence injury (**Figure S1**). The UV-B light had a peak intensity of 0.14W/m^2^ at 312 nm.

### 2.2 Preparation of inoculum

*B. cinerea* B05.10 was grown on malt extract agar (MEA) as described by Zhang et al. (2016). Conidia were harvested from plates by flooding cultures with 20 mL sterile Milli-Q (MQ) water and scraping them with a sterile spatula. The suspension containing mycelium and conidia was filtered through glass wool into a 50 mL tube. Subsequently, spore suspension was washed by centrifugation at 1000 RPM/180 RCF for 10 minutes. The supernatant was discarded and conidia were resuspended in 30-40 mL sterile MQ water. Conidia were counted using a haemocytometer and spore density was adjusted to 1x10^7^ spores/mL water.

Gamborg B5 medium (Duchefa, The Netherlands) contains 3.2 g/L minerals and vitamins (Gamborg et al., 1976) supplemented with sucrose at two concentrations (10 mM or 50 mM) and with potassium phosphate pH 6.0 (10 mM or 50 mM). Potato Dextrose Broth (PDB, Oxoid, UK) medium was prepared at 12 g/L. Inoculum with four spore densities ranging from 30-1000 spores/µL were prepared by dilution in the medium from the stock solution.

### 2.3 Inoculation assay

Detached leaf inoculations were done essentially as described by ten Have et al. (2007). Fully expanded compound leaves were detached and placed with their petioles in wet florist foam in closed trays under high humidity. Inoculum with different spore densities and media was prepared as mentioned above. Droplets of 2 μL were pipetted on the adaxial surface. Four main leaflets from one compound leaf were inoculated with 6 to 8 droplets per leaflet. Disease parameters were recorded from 2 to 4 days post inoculation (dpi) depending on the experiment. Lesion diameter was measured with a digital caliper and lesion areas were measured using Image J software 1.53e (NIH, USA).

### 2.4 *In vitro* germination assay

Spores of *B. cinerea* B05.10 were diluted in media with different sucrose and phosphate concentrations to 1000 spores/µL as used for inoculation assay. 8 droplets of spore suspensions prepared in each medium were placed on glass slides and then incubated under high humidity. Germination of spores was observed under a microscope after 4 h and 6 h and images were taken for analyzing germination rates and length of germ tubes.

### 2.5 Statistical analysis

**Figure 1:**
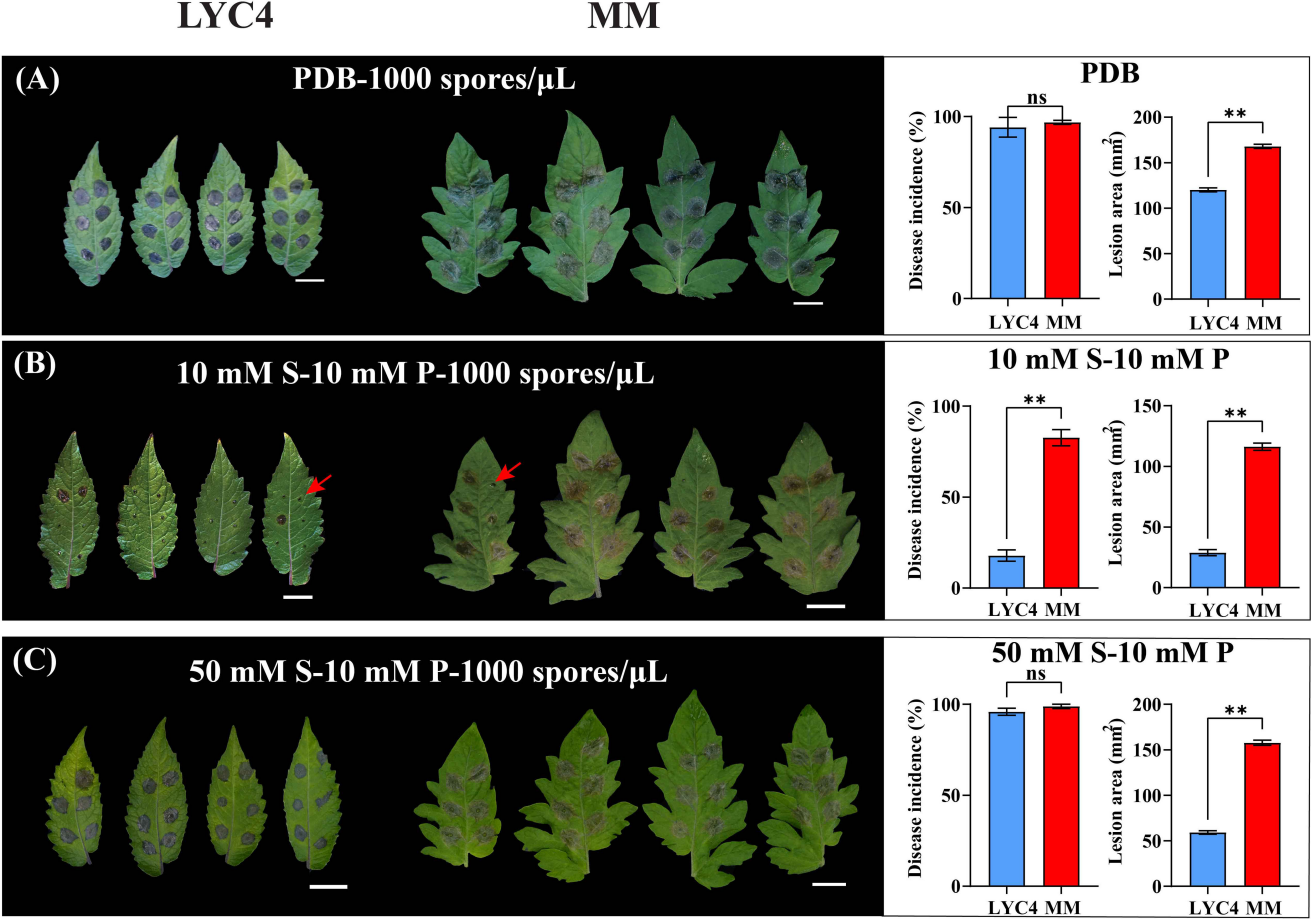
Effect of different media on *B. cinerea* infection on MM and LYC4. Symptoms and disease parameters at 3 dpi from detached leaves inoculated with 2 µL B05.10 inoculum containing 1000 spores/µL either in PDB **(A)**, in GB5 medium supplemented with 10 mM sucrose **(B)**, or GB5 medium supplemented with 50 mM sucrose **(C)**. The scale bar indicates 2 cm. The disease incidence is displayed as the proportion of inoculation droplets forming expanding lesions. The lesion area was measured with ImageJ software. Disease incidence and lesion area are displayed as mean values ± SEM of all measurements from at least three independent inoculation assays. The asterisks represent statistically significant differences determined by student’s t-test (** p-value < 0.01). ns indicates no significant difference. S, sucrose; P, phosphate.

**Figure 2:**
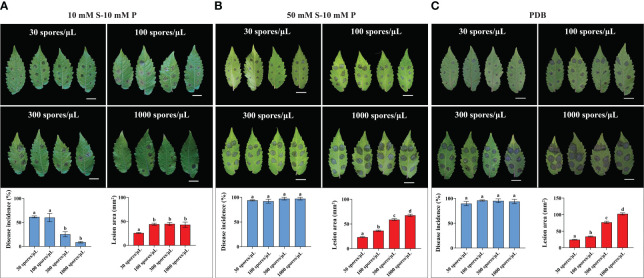
Effect of spore densities on *B. cinerea* infection on LYC4. Symptoms and disease parameters at 3 dpi from detached leaves inoculated with 2 µL B05.10 inoculum containing 30 spores/μL, 100 spores/μL, 300 spores/μL and 1000 spores/uL either in 10 mM sucrose **(A)**, 50 mM sucrose **(B)** or PDB **(C)**. Data are displayed as mean values ± SEM of all measurements from three independent inoculation assays. Different letters indicate statistically significant differences. The scale bar indicates 2 cm. S, sucrose; P, phosphate.

Statistical analysis was performed using GraphPad Prism 9.3.1. Student’s/unpaired T-test was applied to analyze the disease parameters between LYC4 and MM in **Figure 1** and **Supplementary Figure S2**. Differences with two-tailed p-values less than 0.05 and 0.01 were considered statistically significant and were represented by one asterisk (*) and two asterisks (**), respectively. The two-tailed p-values more than 0.05 indicate non-significant differences and were represented by ns. One-way ANOVA with Tukey *post hoc* analysis was performed to compare the disease parameters under multiple infection conditions in **Figure 2** and **Supplementary Figure S3**. Significant differences were indicated by different superscript letters.

### 2.6 Microscopic analysis

Calcofluor white was used to visualize the fungus according to Rasconi et al. (2009). Images were obtained with a Nikon Eclipse 90i fluorescence microscope using DAPI filter (exitation:340-380 nm, dichroic mirror: 400 nm, barrier wavelength: 435-485 nm). 3,3’-Diaminobenzidine (DAB)staining was used to visualize the ROS production as described by Asselbergh et al. (2007) with modifications. Leaf discs were immersed in 5 ml of the freshly prepared DAB solution (1 mg/mL) dissolved in MQ water (pH 3.8 with HCl). Samples were vacuum infiltrated 1-2 times with DAB solution and then immediately destained in ethanol to remove chlorophyll. Toluidine Blue (TB) solution (0.05% in MQ water) was used to visualize both plant cell wall fortifications and the fungal hyphae. Leaf samples were mounted on microscope slides in 50% glycerol. Bright-field images were taken using a binocular as well as a light microscope.

## 3 Results

### 3.1 Exploration of the incompatible interaction between *Botrytis cinerea* and LYC4

Because *B. cinerea* inoculation typically requires nutrient supply in the inoculum to initiate infection, the initial experiments made use of inoculum in PDB which is commonly used in *B. cinerea* infection. Most of the leaf inoculations on LYC4 caused expanding lesions even though the lesions were consistently smaller than on MM (**Figure 1A**). We considered that the strong infection-promoting effect of PDB, because of its abundance in nutrients, might hamper the detection of differences in susceptibility to *B. cinerea* between LYC4 and MM. Thus, we tested a synthetic medium composed of GB5, sucrose and phosphate which allows to control the concentration of components to manipulate the disease progress of *B. cinerea* during artificial infection.

In contrast to the high incidence of expanding lesions (referred to as “compatible interaction”) resulting from inoculation in PDB, spore suspensions in GB5 supplemented with 10 mM sucrose and 10 mM potassium phosphate, caused a higher proportion of non-expanding lesions (**Figure 1B**). Most of these primary lesions (>80%) did not expand even after prolonged incubation, leading to an “incompatible interaction”. When the sucrose concentration in the inoculum was increased from 10 mM to 50 mM, however, the proportion of expanding lesions on LYC4 increased to >90% (**Figure 1C**). The same inoculation conditions on MM leaves resulted in a high proportion of expanding lesions (**Figures 1A-C**), regardless of the medium. The lesion sizes resulting from inoculation in medium with 50 mM sucrose were always larger than with 10 mM sucrose, both in LYC4 (approximately 2-fold) and MM (~50%) (**Figures 1B, C**).

Besides the sucrose concentration, we also tested the effect of adjusting the phosphate concentration from 10 mM to 50 mM (**Supplementary Figure S2**). In a medium containing 10 mM sucrose, the increase of phosphate from 10 to 50 mM resulted in an increase of the proportion of expanding lesions (from ~20% to ~60%), while the sizes of lesions that expanded were not significantly different between the phosphate concentrations (**Figure S2A**). In the medium containing 50 mM sucrose, an increase in phosphate from 10 to 50 mM neither affected the proportion of expanding lesions nor the lesion sizes (**Figure S2B**).

We also analyzed the effect of initial spore densities (30, 100, 300 and 1000 spores/µL, respectively) on the outcome of *B. cinerea* infection on LYC4. When the inoculation medium containing GB5-10 mM sucrose-10 mM phosphate was used, 1000 spores/µL exhibited low disease incidence on LYC4. Reducing the spore density to 30 spores/µL or 100 spores/µL significantly increased the proportion of expanding lesions (**Figure 2A**). However, inoculation with 30 spores/µL resulted in significantly smaller lesions than for the higher spore densities (**Figure 2A**). *B. cinerea* inoculation with the four spore densities in GB5-50 mM sucrose-10 mM phosphate resulted in high (>90%) incidence of expanding lesions without statistical differences (**Figure 2B**). However, lower spore densities (30 and 100 spores/µL) resulted in substantially smaller lesions than inoculation with high spore densities (300 and 1000 spores/µL) (**Figure 2B**). Inoculation with different spore densities in PDB had similar effects as for GB5-50 mM sucrose-10 mM phosphate (**Figure 2C**).

We examined the effect of different sucrose and phosphate concentrations on the germination rate of *B. cinerea* spores and germ tube length *in vitro* (**Figure S3**). In all three media tested the germination rate was ~30% after 4h incubation and approached 100% after 6h. The average germ tube length was slightly but significantly shorter in medium containing 10 mM sucrose and 10 mM phosphate, as compared to medium containing either 50 mM sucrose or 50 mM phosphate (**Figure S3**).

### 3.2 Microscopic analysis of compatible interactions and incompatible interactions

**Figure 3:**
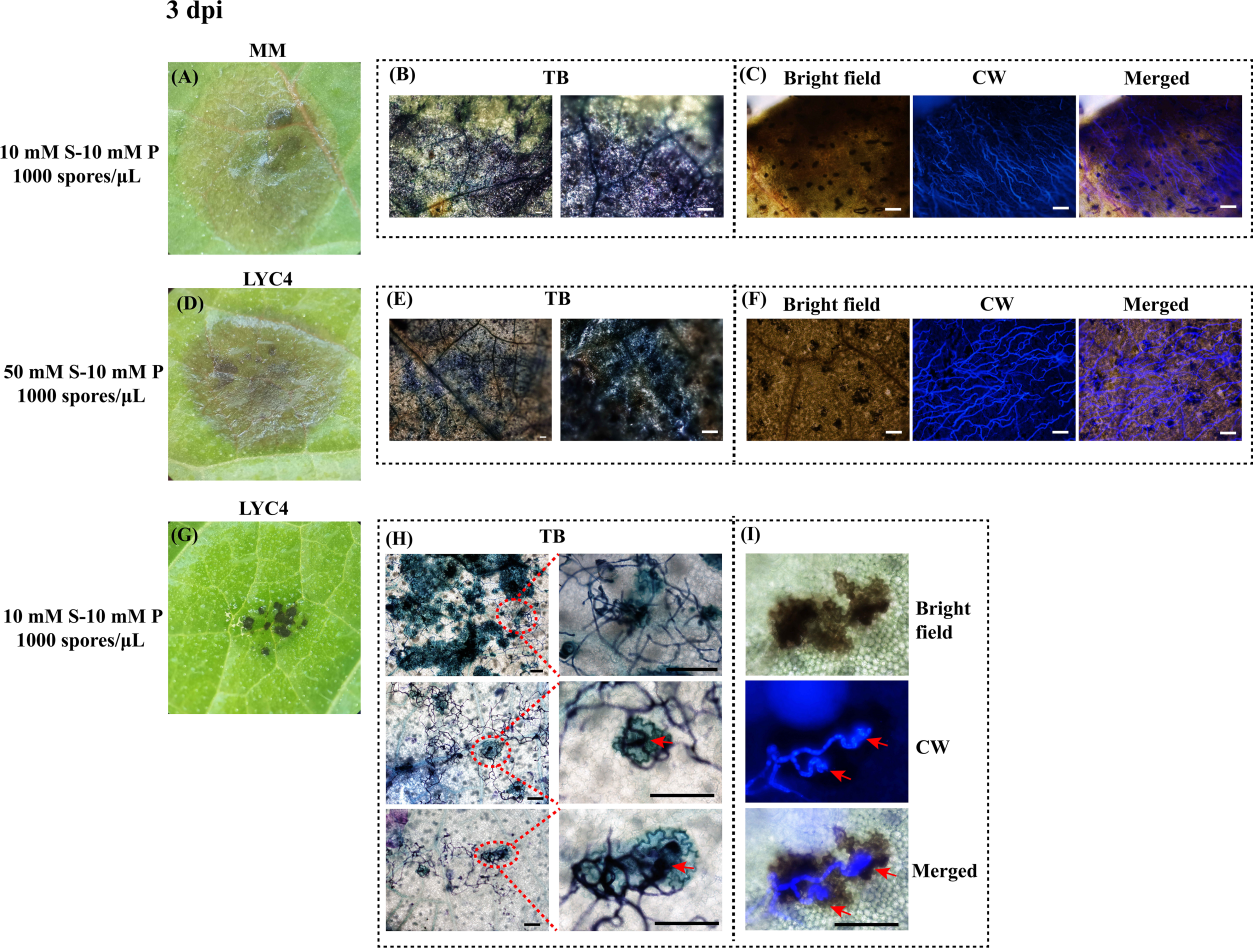
Microscopic analysis of *B. cinerea* infection on MM and LYC4 leaves at 3 dpi. Expanding lesions on MM **(A)** and LYC4 **(D)** as compared with non-expanding lesions on LYC4 **(G)**; Visualization of fungal mycelia and associated plant cell wall fortification by toluidine blue (TB) staining of expanding lesions on MM leaves **(B)** and LYC4 leaves **(E)**, as compared with three separate regions displaying non-expanding lesions on LYC4 leaves **(H)**; Visualization of fungal mycelia by calcofluor white staining under DAPI filter of expanding lesions on MM leaves **(C)** and LYC4 leaves **(F)**, as compared with incompatible interaction on LYC4 (**I**, consisting of a bright field image, a DAPI-filter image and the merged image). Scale bar indicates 100 μM. Red arrow indicates fungal penetration by appressorium. S, sucrose; P, phosphate.

Inoculation of *B. cinerea* on MM or LYC4 under conditions that resulted in compatible interactions developed large lesions of rotten leaf tissue at 3 dpi (**Figures 3A, D**). Staining the samples with Toluidine Blue resulted in massive staining of plant and fungal tissue, with no distinct structures being revealed (**Figures 3B, E**) and calcofluor white staining of the sample detected *B. cinerea* hyphae profusely growing in the lesion area (**Figures 3C, F**). In stark contrast to the brown, water-soaked expanding lesions on MM, non-expanding lesions on LYC4 (obtained from inoculation in GB5-10 mM sucrose-10 mM phosphate) most frequently appeared as tiny (100-200 μm), dark black and scattered necrotic dots, restricted to a part of the area of the inoculation droplet (**Figure 3G**). Staining of incompatible interactions on LYC4 at 3 dpi by Toluidine Blue revealed staining of clusters of epidermal cells (**Figure 3H**). Enlargement of the images in panel 3H revealed the presence of fungal hyphae with appressoria (indicated by red arrows) on top of these clusters of Toluidine Blue-stained plant cells, which resemble “microlesions” as described by Porquier et al. (2021). Calcofluor white staining of the incompatible interactions on LYC4 detected fungal hyphae on the leaf surface that produced appressorium-like penetration structures (indicated by red arrows) but hyphae were not detected inside the host tissue (**Figure 3I**).

**Figure 4:**
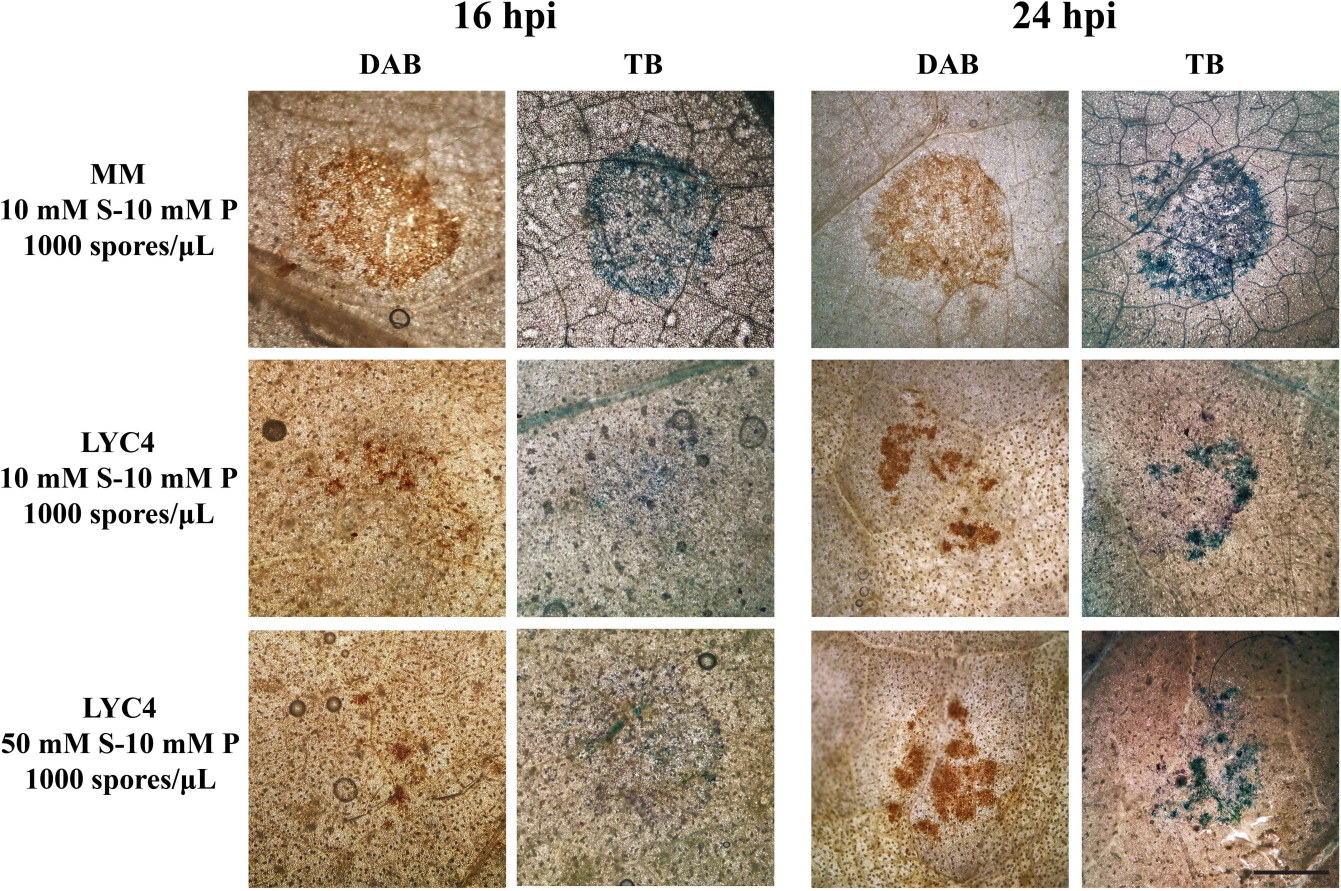
Plant defense responses on MM and LYC4 under different inoculation conditions were visualized by DAB and Toluidine Blue (TB) staining at 16 hpi and 24 hpi in the area of 2 µL inoculum droplet. The scale bar indicates 1 mm. S, sucrose; P, phosphate.

Microscopic analysis of plant defense responses was performed using DAB staining to visualize ROS production and Toluidine Blue staining to reveal plant cell wall fortification in the early stages (16 and 24 hpi) of compatible interactions on MM and LYC4, as well as incompatible interactions on LYC4 (**Figure 4**). Plant defense responses in MM occurred homogeneously in most of the epidermal cells under the entire inoculum droplet (**Figure 4**, top row). By contrast, *B. cinerea* infection on LYC4 using two different inoculation media (1000 spores/μL in 10 mM sucrose medium or in 50 mM sucrose medium, resulting in incompatible and compatible interactions, respectively) elicited limited plant responses in dispersed patches of epidermal cells within the droplet area at 16 and 24 hpi. In LYC4 inoculations, we neither observed obvious differences between the two timepoints, nor between the two inoculation media, resulting in opposite infection outcomes. These observations suggested a distinct spatial pattern and amplitude of defense responses in LYC4 as compared to MM, however, it remains unclear how these responses relate to the resistance mechanism in LYC4.

## 4 Discussion

Quantitative resistance to *B. cinerea* was identified in several wild relatives of tomato (*Solanum lycopersicum*), including *Solanum habrochaites* LYC4 (ten Have et al., 2007). At least ten QTLs were identified in *S. habrochaites* (Finkers et al., 2007a; Finkers et al., 2007b) but the mechanisms that contribute to this resistance remain to be unraveled. We focused on the characterization of leaf resistance based on detached leaf inoculation assays under lab conditions. When inoculum was prepared with PDB medium almost all the inoculation droplets on LYC4 caused expanding lesions and fully colonized the leaflets after 4 days. Nevertheless, the average size of lesions on LYC4 leaves at all time points after inoculation was significantly smaller than that on MM. We presumed that the nutrient-rich PDB medium was somehow favorable for *B. cinerea*. Host resistance might be overwhelmed by the aggressiveness of the fungus and as a consequence, potential differences in disease development between a susceptible and a (partially) resistant genotype would be masked. Derckel et al. (1999) reported a study of the interaction of *B. cinerea* with grape, in which the inoculation with the less aggressive strain T4 enhanced the production of defense compounds, whilst defense responses were significantly compromised or delayed upon inoculation with the more aggressive strain T8. We subsequently used synthetic medium comprising salts, sucrose and phosphate, which allowed us to influence the outcome of fungal infection (Benito et al., 1998). Inoculation of 1000 spores/µL in GB5 medium supplemented with 10 mM sucrose and 10 mM potassium phosphate on LYC4 leaves resulted in high incidence (> 70%) of non-expanding lesions (incompatible interaction), visible as dark black spots under the droplets of inoculum. A similar observation was reported in a different wild tomato relative, *S. lycopersicoides*. Leaf inoculation of *B. cinerea* using the same GB5-sucrose-phosphate medium frequently resulted in confined primary lesions which appeared as dispersed black necrosis (Guimarães et al., 2004). By contrast, under the same inoculation condition, most of the inoculations on MM resulted in expanding lesions defined as compatible interaction (>70%). Remarkably, we observed that *B. cinerea* infection in LYC4 could be promoted by increasing the concentrations of either sucrose or phosphate. The resistance in LYC4 (high incidence of non-expanding lesions) was partly compromised by increasing the phosphate concentration and fully by increasing the sucrose concentration, thus converting the incompatible interaction into a fully compatible interaction. How the sucrose and phosphate concentration in the inoculation droplet facilitates fungal infection after the fungus has penetrated the leaf surface remains to be clarified. We assume that the differences in composition of the inoculum do not influence the gene expression in the host plant, as the inoculation droplet is only 2 μL in size and is applied on the epidermal surface. Our main hypothesis is that a higher sugar concentration either promotes the expression of fungal genes which play important roles in virulence on LYC4, or conversely, reduces (by catabolite repression) the expression of fungal genes that trigger a resistance response in LYC4. In order to test this hypothesis, a study on the transcriptional changes in both fungus and host plant upon *B. cinerea* inoculation of MM and LYC4 with different sucrose concentrations is in progress, and we are generating *B. cinerea* mutants in which catabolite repression is abolished. With regard to the effect of phosphate concentration on disease development, we presume that it relates to the dynamics of the pH profile in the inoculation site. It is well established that *B. cinerea* acidifies its environment and specifically the inoculation droplet during the early phases of infection (Verhoeff et al., 1988; Müller et al., 2018). Not only does the external pH influence the gene expression in *B. cinerea* (Rolland et al., 2009; Müller et al., 2018), it also affects the secretion and the enzyme activity of secreted enzymes that contribute to the infection process (Manteau et al., 2003; Li et al., 2012).

In the present study, spore densities played contrasting roles in *B. cinerea* infection depending on the inoculation conditions. Although inoculation with low spore densities in PDB or in GB5-50 mM sucrose-10 mM phosphate on LYC4 did not significantly affect the proportion of expanding lesions, it significantly reduced the aggressiveness of *B. cinerea* infection manifested as smaller lesions. Counter-intuitively, reduction in spore density in 10 mM sucrose inoculum, significantly increased the proportion of inoculation droplets forming expanding lesions on LYC4, as compared with 1000 spores/µL. How the interplay between spore density and sugar concentration affects the percentage of expanding lesions remains to be further studied.

Since *B. cinerea* is a necrotrophic fungus, it benefits from cell death processes in the host plant, and in fact, the induction of host cell death is essential for *B. cinerea* to cause disease (van Kan, 2006). In the incompatible interaction with LYC4, it is unknown how the development of necrotic microlesions confines the *B. cinerea* infection. If plant cell death per se is crucial, then what is the difference between a plant cell death process that can stop fungal infection (in the incompatible interaction) and a cell death process which facilitates fungal colonization (in the compatible interaction)? It has been proposed that the outcome of *B. cinerea* interaction with its host depends on a balance between distinct programmed cell death (PCD) pathways, autophagy and apoptosis (Veloso and van Kan, 2018). Although activation of either PCD pathway results in plant cell death and necrotic symptoms, the induction of either pathway has opposing consequences for a host (resistance or susceptibility). A mutant from the necrotrophic pathogen *S. sclerotiorum*, a species closely related to *B. cinerea*, triggered autophagic host cell death that resulted in plant resistance (Kabbage et al., 2013). Conversely, *B. elliptica*-induced host cell death displayed features of apoptosis and was shown to be essential for susceptibility (van Baarlen et al., 2007). The manipulation of different infection outcomes (compatible and incompatible) in LYC4 using different inoculation conditions provides a tool to better understand the interaction between plants and necrotrophs.

According to a previous study, resistance to *B. cinerea* in the tomato ABA-deficient mutant *sitiens* was partially conferred by early and localized defense responses (Asselbergh et al., 2007; Curvers et al., 2010). During the incompatible interaction (non-expanding lesions), accumulation of H_2_O_2_ and cell wall fortification were observed in the epidermal cells of *sitiens* between 4 h and 8 h after *B. cinerea* inoculation (Asselbergh et al., 2007), whereas on wild-type leaves, H_2_O_2_ accumulation was observed after 24 h in the mesophyll cell layer and it was followed by spreading lesions (Asselbergh et al., 2007). Furthermore, *B. cinerea* infection on *sitiens* could induce faster and stronger expression of defense-related genes, such as genes encoding pathogenesis-related (PR) proteins. However, in our study, accumulation of H_2_O_2_ and cell wall fortification represented by phenolic deposition during infection were faster and stronger in MM than LYC4. Besides, inoculation with 10 mM sucrose medium or 50 mM sucrose medium on LYC4 did not exhibit notable differences in DAB and toluidine blue staining at least in the first 24 h.

In conclusion, the main deliverable of this work is an experimental protocol by which disease development in the *B. cinerea-*LYC4 interaction can be controlled to either make it compatible or incompatible by simply using different sucrose concentrations in the inoculum. This method provides a convenient tool to study both types of interactions through RNA sequencing. Our next study will describe comparisons of transcriptional changes, in host plants and *B. cinerea*, during compatible and incompatible interactions.

## Data availability statement

The original contributions presented in the study are included in the article/**Supplementary Material**. Further inquiries can be directed to the corresponding author.

## Author contributions

YY and JK designed the study; BE cultivated the plants; YY, SQ and IA-E executed the experiments; YY prepared all figures; YY and JK wrote the manuscript.

## Supplementary material

Supplementary Figure 1 UV light treatment on LYC4 plants to prevent intumescence injury. Intumescence on LYC4 plants growing in the greenhouse causes leaf chlorosis, wilting and senesce **(A-C)**; UV-B light treatment on tomato plants in the greenhouse **(D)**; Prevention of intumescence development on LYC4 leaves by UV-B light treatment **(E)**.

Supplementary Figure 2 Effects of changing phosphate concentration in the inoculation medium on disease development in LYC4 leaves, both for the disease incidence and the lesion size at 3 dpi. **(A)** Effects of changing phosphate concentration from 10 to 50 mM in GB5 medium containing 10 mM sucrose. **(B)** Effects of changing phosphate concentration from 10 to 50 mM in GB5 medium containing 50 mM sucrose. S = sucrose, P = phosphate.

Supplementary Figure 3 Germination of B. cinerea spores in media with three different combinations of sucrose and phosphate concentrations (either 10 or 50 mM as indicated). Representative images taken at 4h and 6h of incubation are shown. The bar graphs provide the germination incidence (%) and the germ tube length (μm). S = sucrose, P = phosphate.
