# Supplementary figures and images for: Leaf resistance to Botrytis cinerea in wild tomato Solanum habrochaites depends on inoculum composition
Source: Front Plant Sci. 2023 Aug 2;14:1156804. doi: 10.3389/fpls.2023.1156804 (PMC10433766; doi:10.3389/fpls.2023.1156804)

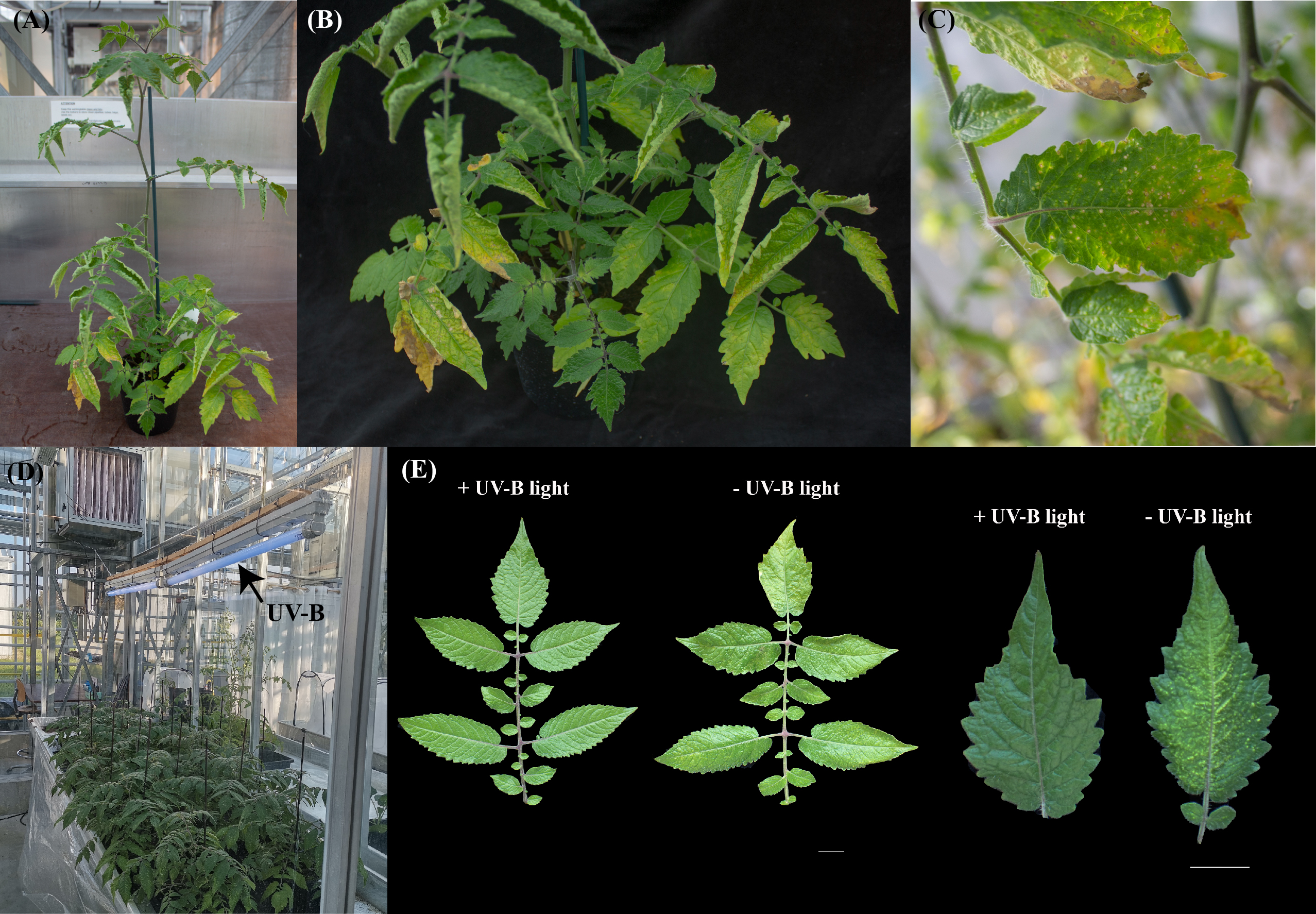

Supplement: Supplementary Figure 1 — UV light treatment on LYC4 plants to prevent intumescence injury. Intumescence on LYC4 plants growing in the greenhouse causes leaf chlorosis, wilting and senesce (A-C); UV-B light treatment on tomato plants in the greenhouse (D); Prevention of intumescence development on LYC4 leaves by UV-B light treatment (E). [file Image_1.jpeg]

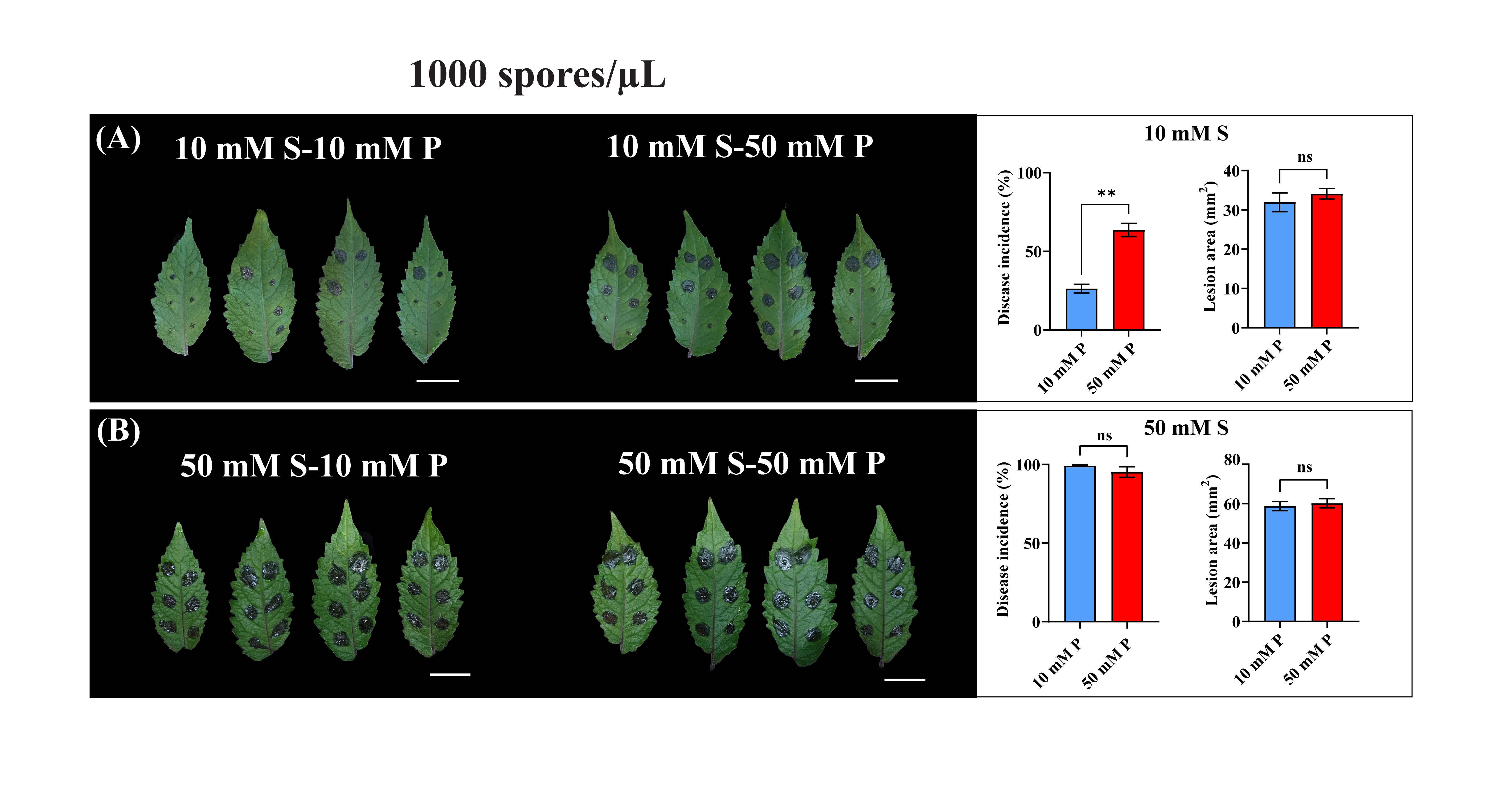

Supplement: Supplementary Figure 2 — Effects of changing phosphate concentration in the inoculation medium on disease development in LYC4 leaves, both for the disease incidence and the lesion size at 3 dpi. (A) Effects of changing phosphate concentration from 10 to 50 mM in GB5 medium containing 10 mM sucrose. (B) Effects of changing phosphate concentration from 10 to 50 mM in GB5 medium containing 50 mM sucrose. S = sucrose, P = phosphate. [file Image_2.jpg]

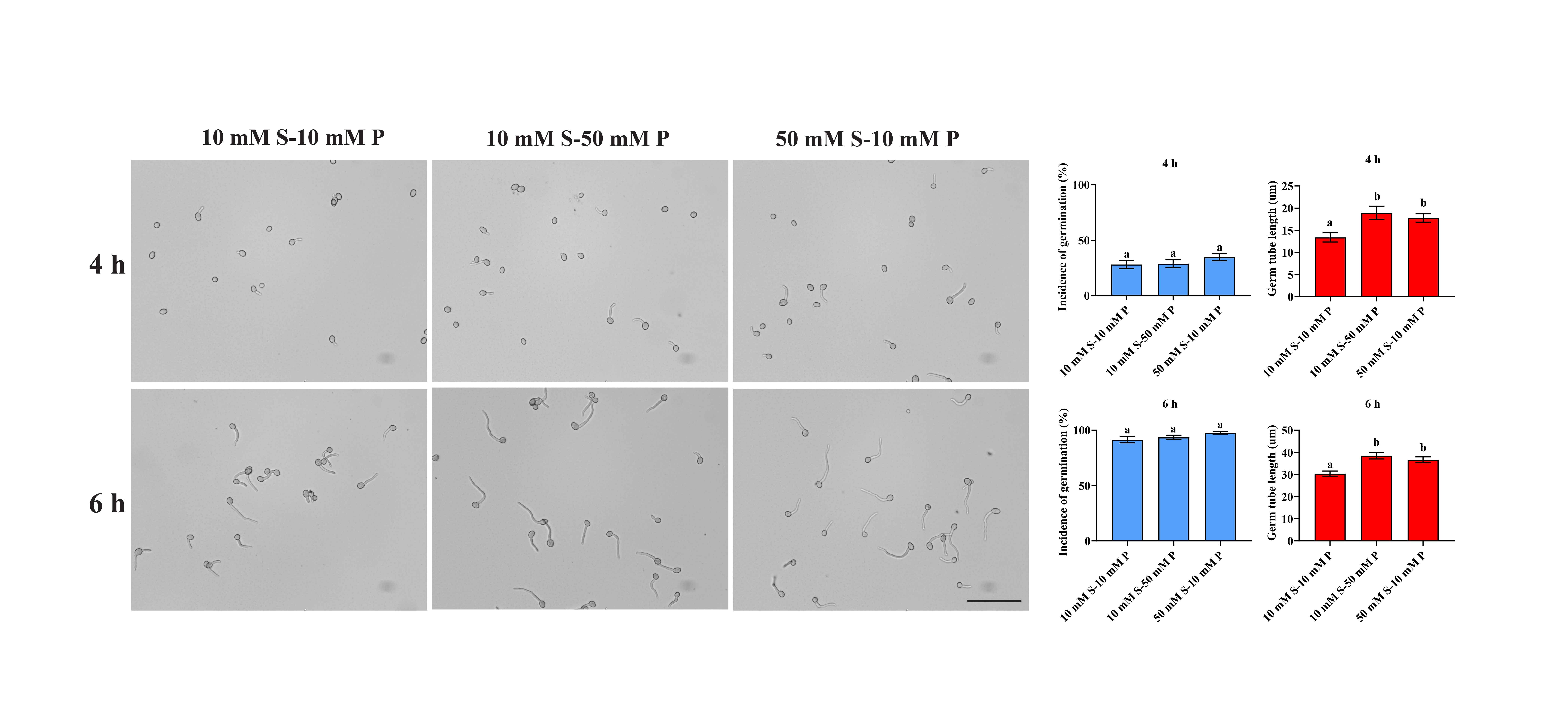

Supplement: Supplementary Figure 3 — Germination of B. cinerea spores in media with three different combinations of sucrose and phosphate concentrations (either 10 or 50 mM as indicated). Representative images taken at 4h and 6h of incubation are shown. The bar graphs provide the germination incidence (%) and the germ tube length (μm). S = sucrose, P = phosphate. [file Image_3.jpeg]
